# Protosappanin B promotes apoptosis and causes G_1_ cell cycle arrest in human bladder cancer cells

**DOI:** 10.1038/s41598-018-37553-z

**Published:** 2019-01-31

**Authors:** Xihua Yang, Lili Zhao, Tingting Zhang, Junfeng Xi, Shuze Liu, Liansheng Ren, Yaqin Zheng, Huanhu Zhang

**Affiliations:** 1 Affiliated Cancer Hospital, Shanxi Medical University, Taiyuan, 030001 China; 2 Research Institute of Applied Biology, Shanxi University, Taiyuan, 030006 China; 3 Department of Computer Science, Rensselaer Polytechnic Institute, Troy, 12180 USA

## Abstract

The aim of the study was to investigate the effects of protosappanin B on the proliferation and apoptosis of bladder cancer cells. The effects of protosappanin B (12.5, 25, 50, 100, or 200 μg/mL, 48 h) on proliferation of SV-HUC-1, T24 and 5637 cells was assessed using the MTT assay. The effects of protosappanin B (100, 150, 200, 250, or 300 μg/mL, 48 h) on cell apoptosis and cell cycle were analyzed using flow cytometry. T24 and 5637 cells treated with 200 µg/mL protosappanin B showed morphological changes (shrinkage, rounding, membrane abnormalities, and reduced adhesion), but protosappanin B had no proliferation arrest effect on SV-HUC-1 cells. Protosappanin B caused concentration-dependent inhibition of cell growth, with IC_50_ of 82.78 µg/mL in T24 cells and 113.79 µg/mL in 5637 cells. Protosappanin B caused concentration-dependent increases in T24 and 5637 cell apoptosis (100–300 µg/mL). The effects of protosappanin B on the cell cycle in both cell types was G_1_ arrest with reductions in the proportion of S-phase cells and proliferation index. A proteomics analysis showed that protosappanin B modulated a number of genes involved in the cell cycle. In conclusion, protosappanin B inhibits the proliferation and promotes the apoptosis of T24 and 5637 human bladder cancer cells in a concentration-dependent manner, possibly via interference with cell cycle regulation, preventing G_1_-to-S transition.

## Introduction

Bladder cancer is one of the most common malignant tumors, ranked eleventh among malignant cancers in terms of incidence^1^, and is associated with high mortality^1^. It has been estimated that, in 2012, around 430,000 new cases of bladder cancer occurred worldwide and over 165,000 people died from it^2^. Bladder cancer affects men more commonly than women, and smoking is recognized as an important risk factor^3^. The incidence of bladder cancer in China during the last 10 years has shown an increasing trend both in urban and rural areas, and this may be associated with the increases in tobacco consumption, level of industrialization, and population aging^4^. Bladder transitional cell carcinoma is the most frequent type, accounting for 95% of the cases. Around 30% of patients with bladder cancer present with an invasive form of the disease associated with a high risk of metastasis^5^. Various strategies are currently available for the management of bladder cancer, including transurethral resection of bladder tumor (TURBT), radical cystoprostatectomy, radiotherapy, chemotherapy, and intravesical therapy^5^. Among these, the main treatment approaches both in China and abroad is surgery combined with intravesical chemotherapy. There have been several recent advances in the diagnosis and treatment of bladder cancer^6^, including research on new targeted therapies^7^. Nevertheless, the available surgical and medical therapies are associated with significant adverse effects on the quality of life and with high recurrence and mortality rates^2^. In particular, the chemotherapeutic drugs (methotrexate, vincristine, doxorubicin, cisplatin, and cytosine) and biological therapies (BCG, immunologic and inactivated bacterial solutions) currently used in clinical practice are associated with high costs, significant adverse effects, and various complications^8^. These limitations highlight the need to develop novel treatment approaches.

Traditional Chinese medicine (TCM) has a long history in the treatment of cancer, with many components of TCMs being reported to have anti-cancer properties^9^. With the increasing application of molecular biology in oncology research, there has been considerable interest in studying the anti-tumor effects of TCMs and identifying the responsible compounds and possible underlying mechanisms. Lignum Sappan, derived from the heartwood of *Caesalpinia sappan* L., is commonly used in TCM and promotes blood circulation for removing obstruction in collaterals. In addition to anti-inflammatory^10^, anti-allergy^11^, anti-fungal^12^, anti-viral^13^, anti-oxidative^14^, and vasorelaxant^15^ properties, Lignum Sappan has also been shown to have anti-cancer effects. Indeed, Lignum Sappan extracts have been reported to reduce the viability of a wide variety of cancer cells^16^, including head and neck^17^, sarcoma^18^, hepatocellular carcinoma^18^, lung adenocarcinoma^18^, colorectal adenocarcinoma^18^, gastric cancer^19^, leukemia^20^, and ovarian cancer^21^ cell lines. Lignum Sappan has also been shown to inhibit tumor growth *in vivo* in a mouse xenograft model bearing S180 sarcoma cells^18^.

In recent years, there has been considerable interest in identifying the active components of Lignum Sappan and studying the mechanisms by which these components inhibit tumor growth. Brazilin is an important active component of Lignum Sappan and has been found to exert an anti-cancer effect. Brazilin has been shown to inhibit the proliferation of human bladder cancer T24 cells^22^ and induce the apoptosis of multiple myeloma U266 cells^23^, glioma U87 cells^24^, sarcoma S180 cells^18^, hepatocellular carcinoma HepG2 cells^18^, lung adenocarcinoma H522 cells^18^, colorectal adenocarcinoma Colo205 cells^18^, and head and neck squamous cell carcinoma Cal27 cells^25^.

Protosappanin B is another major component of Lignum Sappan and is listed by the Chinese Pharmacopoeia^26^ as an indicator of the quality of Lignum Sappan preparations. At present, there are very few published studies describing the effects of protosappanin B. Anti-inflammatory^27^, anti-bacterial^28^, and anti-oxidative^29^ properties of protosappanin B have been reported, and pharmacokinetic and bioavailability studies have been conducted in rodents^30,31^. Protosappanin B has also been shown to protect PC12 cells against apoptosis induced by oxygen-glucose deprivation^32^. Nevertheless, very few studies have investigated the anti-cancer effects of protosappanin B. In a recent study, protosappanin B was shown to reduce the viability of human bladder cancer T24 cells and mouse bladder cancer BTT cells and inhibit the proliferation of two human colon cancer cell lines (HCT-116 and SW-480)^33^. In addition, protosappanin B was found to inhibit tumor formation in, and improve the survival of, tumor-bearing mice^33^.

As shown by a previous study by our group, protosappanin B inhibits the growth of bladder cancer cells^33^, but the doses and the mechanisms involved are still poorly understood. Therefore, the aims of the present study were to investigate the effects of protosappanin B on the proliferation and apoptosis of two bladder cancer cell lines cultured *in vitro* and to explore the potential mechanisms for the observed effects using a proteomics analysis. The cell lines chosen for the experiments were T24, representing invasive bladder cancer, and 5637, representing high-risk superficial bladder cancer.

## Results

### Effects of protosappanin B on cell morphology

**Figure 1:**
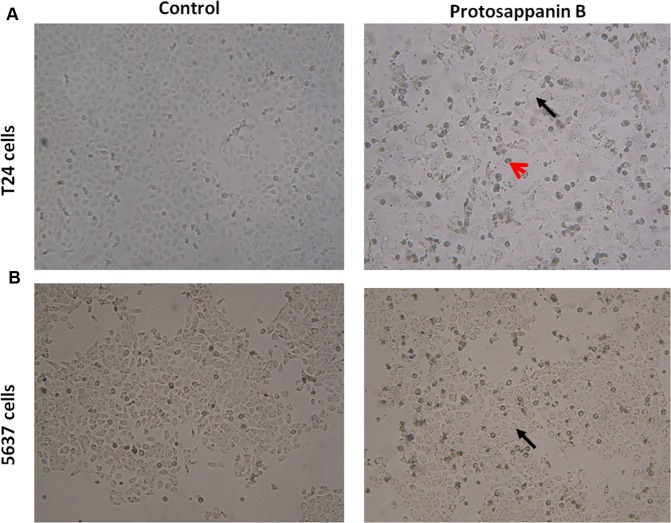
Effects of protosappanin B on cell morphology. (**A**) T24 cells observed under light microscopy (×100). Left panel: control cells. Right panel: T24 cells treated with 200 μg/mL protosappanin B. (**B**) 5637 cells observed using light microscopy (×100). Left panel: control cells. Right panel: 5637 cells treated with 200 μg/mL protosappanin B. Black arrows: cell debris. Red arrow: irregular shape cell.

T24 cells in the control group were spindle-shaped and substantial in volume. The cells had large nuclei, an abundance of cytoplasm and numerous cellular extensions. The cells were also well attached to the wall (Fig. 1A). In contrast, T24 cells treated with 200 μg/mL protosappanin B were irregular in shape and smaller and more rounded than untreated cells (Fig. 1A). In addition, the cell boundary was not smooth, and the cells were more loosely attached to the wall (Fig. 1A). Cell debris due to cell lysis was also observed (Fig. 1A). The 5637 cells in the control group were oval-shaped, with abundant cytoplasm and numerous cellular extensions, and the cells were well attached to the wall (Fig. 1B). Protosappanin B-treated 5637 cells were smaller, more rounded, and more loosely attached to the wall (Fig. 1B). The cell surface was not smooth and showed evidence of blebbing; in addition, there was lysis of some cells (Fig. 1B).

### Effects of protosappanin B on cell growth and proliferation

Protosappanin B (12.5–200 µg/mL) caused a concentration-dependent inhibition of T24 cell growth and proliferation (Table 1), with growth inhibition rates of approximately 31% at 100 µg/mL and 92% at 200 µg/mL (*P* < 0.05). Protosappanin B (50–200 µg/mL) also caused a concentration-dependent inhibition of 5637 cell growth (Table 1). The growth inhibition rates were around 21% at 100 µg/mL and 76% at 200 µg/mL (*P* < 0.05). Protosappanin B (50–200 µg/mL) also caused a concentration-dependent inhibition of SV-HUC-1 cell growth (Table 1). The growth inhibition rates were about 14.56% at 100 µg/mL and 56.54% at 200 µg/mL (*P* < 0.05), but the inhibitory rate was lower than that in cancer cells. The IC_50_ values for the inhibition of cell growth by protosappanin B (applied for 48 h) were 82.78 µg/mL in T24 cells and 113.79 µg/mL in 5637 cells.

**Table 1:** Inhibition of the growth of T24, 5637 and SV-HUC-1cells by protosappanin B.

Protosappanin B concentration	T24 cells		5637 cells		SV-HUC-1	
	OD value	Inhibition rate (%)	OD value	Inhibition rate (%)	OD value	Inhibition rate (%)
0 µg/mL (control)	0.890 ± 0.013	—	1.077 ± 0.015	—	0.886 ± 0.016	—
12.5 µg/mL	0.745 ± 0.045*	16.29	0.982 ± 0.060	8.82	0.833 ± 0.025	5.98
25 µg/mL	0.736 ± 0.013*	17.30	0.921 ± 0.085	14.48	0.786 ± 0.024	11.29
50 µg/mL	0.724 ± 0.030*	18.65	0.919 ± 0.029*	14.67	0.757 ± 0.035*	14.56
100 µg/mL	0.616 ± 0.039*	30.79	0.847 ± 0.047*	21.36	0.726 ± 0.021*	18.06
200 µg/mL	0.070 ± 0.033*	92.13	0.260 ± 0.072*	75.86	0.385 ± 0.028*	56.54

Data shown as means ± standard deviation (n = 5). OD: optical density. *P < 0.05 versus the control group.

### Effects of protosappanin B on cell apoptosis

**Figure 2:**
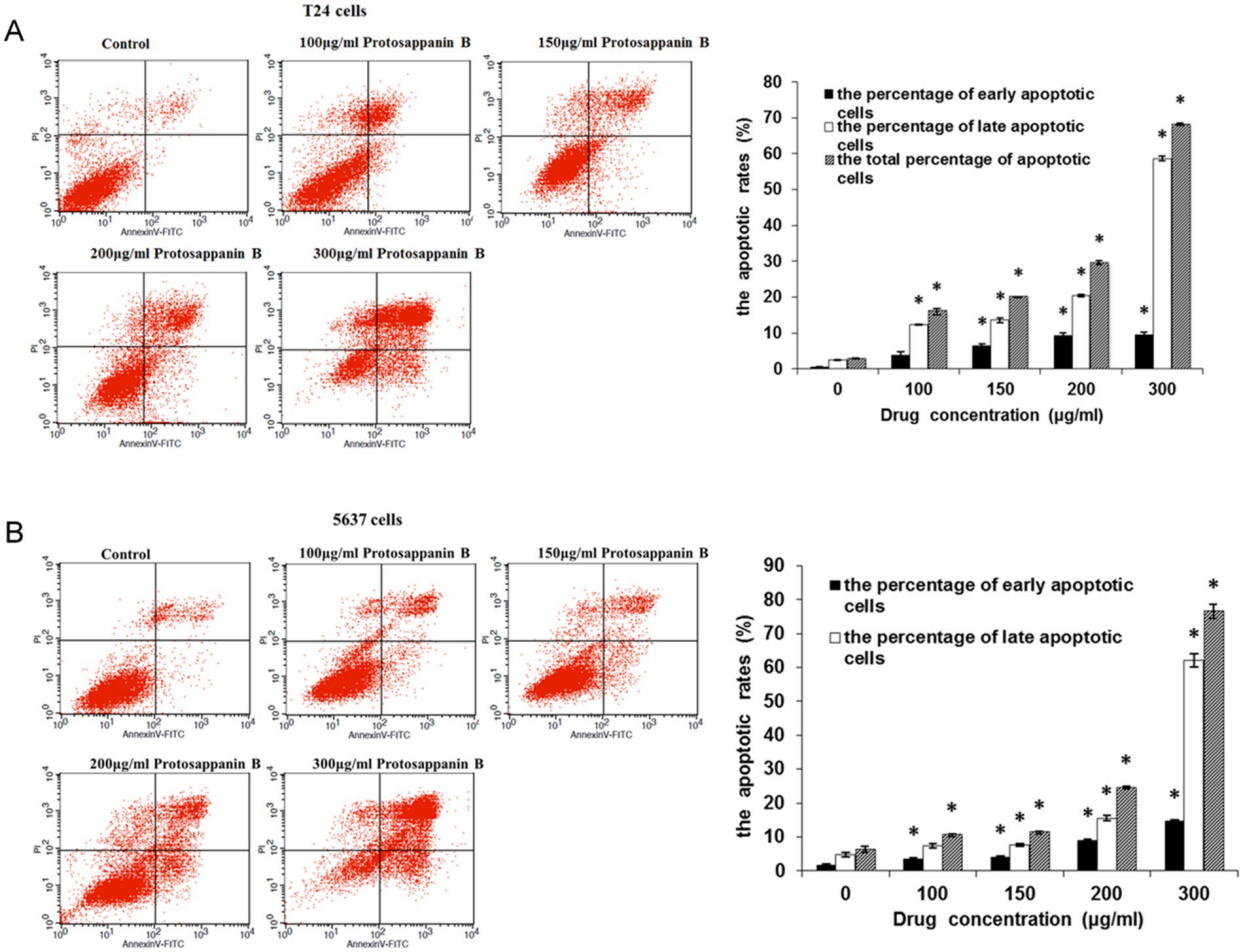
Effects of protosappanin B on cell apoptosis. (**A**) Apoptosis of T24 cells. (**B**) Apoptosis of 5637 cells. Apoptosis was measured using the annexin-V/propidium iodide (PI) assay. The scatterplots illustrate the effects of various concentrations (100–300 µg/mL) of protosappanin B. Normal live cells (annexin-V-negative and PI-negative) are shown in the lower left quadrant, early apoptotic cells (annexin-V-positive and PI-negative) in the lower right quadrant, and late apoptotic cells (annexin-V-positive and PI-positive) in the upper right quadrant. Mean data showing the concentration-dependent effects of protosappanin B on cell apoptosis are presented in the bar graphs. *P < 0.05.

**Figure 3:**
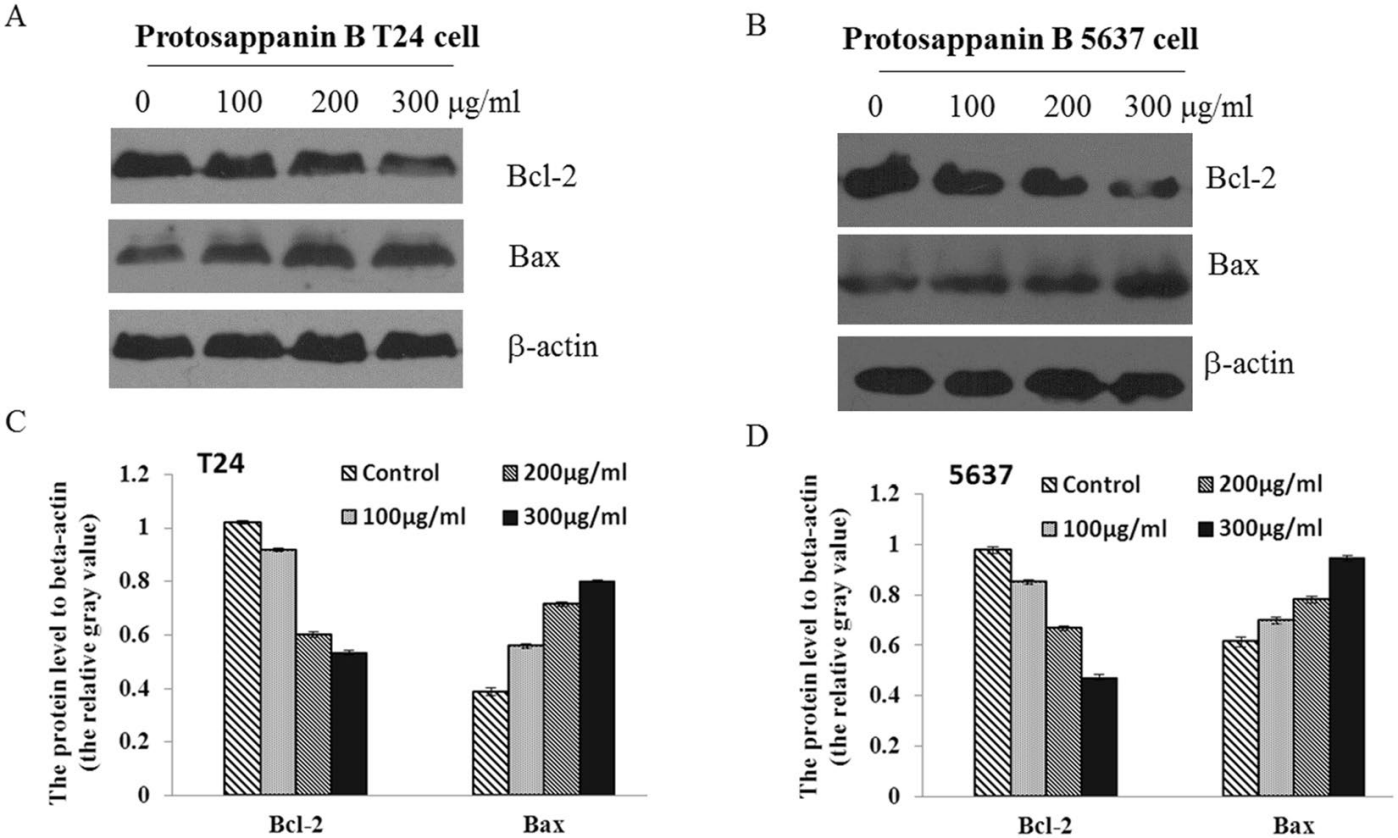
Effects of protosappanin B on Bax and Bcl-2 expression. (**A**,**B**) Representative western blots. (**C**) Effect of protosappanin B (0, 100, 200, and 300 µg/mL) on the protein expression of Bcl-2 and Bax in T24 cells. (**D**) Effect of protosappanin B (0, 100, 200, and 300 µg/mL) on the protein expression of Bcl-2 and Bax in 5637 cells.

As illustrated in Fig. 2A, protosappanin B caused a concentration-dependent increase in the number of early apoptotic cells (150–300 µg/mL; *P* < 0.05) and late apoptotic cells (100–300 µg/mL; *P* < 0.05). The total numbers of apoptotic cells were also increased in a concentration-dependent manner (100–300 µg/mL; *P* < 0.05). Protosappanin B exerted similar concentration-dependent effects in 5637 cells (Fig. 2B), increasing the number of early apoptotic cells (100–300 µg/mL; *P* < 0.05) and late apoptotic cells (150–300 µg/mL; *P* < 0.05), as well as the total number of apoptotic cells (100–300 µg/mL; *P* < 0.05). As shown in Fig. 3, protosappanin B decreased the expression of Bcl-2 (100–300 µg/mL; *P* < 0.05) and increased the expression of Bax (100–300 µg/mL; *P* < 0.05) in a concentration-dependent manner in T24 and 5637 cells. These results indicate that protosappanin B could induce bladder cancer cell apoptosis.

### Effects of protosappanin B on the cell cycle

**Figure 4:**
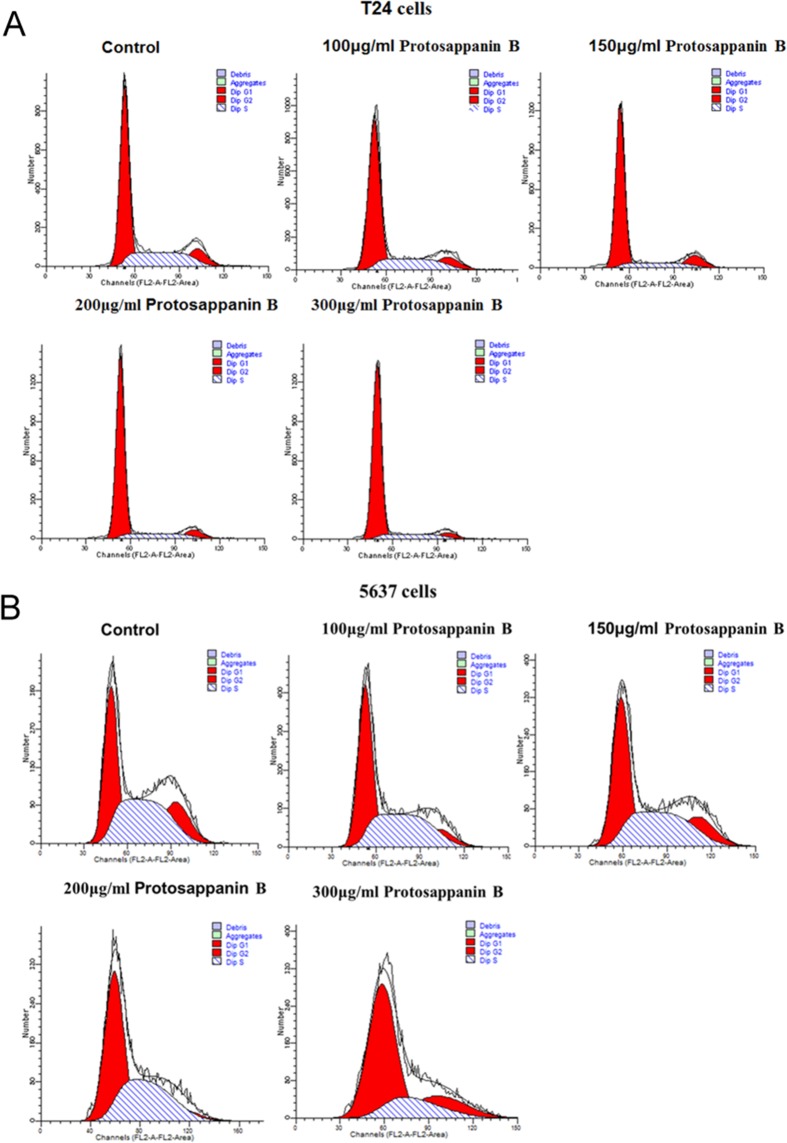
Effects of protosappanin B on the cell cycle. (**A**) T24 cells. (**B**) 5637 cells. Cells were treated with a range of concentrations of protosappanin B (0–300 µg/mL), and cell cycle analysis was undertaken using a commercial kit.

For both T24 cells (Fig. 4A and Table 2) and 5637 cells (Fig. 4B and Table 3), protosappanin B (applied for 48 h) caused a concentration-dependent decrease in the proportion of cells in the S phase or G_2_ phase and a corresponding increase in the proportion of cells in the G_1_ phase. Therefore, the effect of protosappanin B in both cell types was to block the cells in G1. At 300 µg/mL protosappanin B, the proliferative index was decreased significantly from 37.04 ± 3.11 to 17.26 ± 1.71 (*P* < 0.05) in T24 cells and from 62.57 ± 3.03 to 35.41 ± 3.38 in 5637 cells (*P* < 0.05; Tables 2 and 3).

**Table 2:** Effect of protosappanin B on the cell cycle in T24 cells.

Protosappanin B concentration	G_1_ phase	S phase	G_2_ phase	Proliferative index
0 µg/mL (control)	62.96 ± 3.10	26.28 ± 2.03	10.76 ± 1.08	37.04 ± 3.11
100 μg/ml	64.97 ± 1.03	23.58 ± 0.16	11.46 ± 0.93	35.03 ± 1.03
150 μg/ml	73.92 ± 0.88*	14.40 ± 0.73*	11.68 ± 0.32	26.08 ± 0.87*
200 μg/ml	79.80 ± 0.80*	13.46 ± 0.13*	6.75 ± 0.72*	20.21 ± 0.80*
300 μg/ml	82.74 ± 1.71*	12.85 ± 1.62*	4.41 ± 0.42*	17.26 ± 1.71*

Data shown as means ± standard deviation (n = 3). OD: optical density. *P < 0.05 versus the control group.

**Table 3:** Effect of protosappanin B on the cell cycle in 5637 cells.

Protosappanin B concentration	G_1_ phase	S phase	G_2_ phase	Proliferative index
0 µg/mL (control)	37.43 ± 3.03	42.24 ± 3.89	20.33 ± 0.89	62.57 ± 3.03
100 μg/ml	45.76 ± 3.06*	41.46 ± 3.32	12.78 ± 2.63	54.24 ± 3.06*
150 μg/ml	45.23 ± 2.35*	40.03 ± 2.33	14.74 ± 2.91	54.77 ± 2.35*
200 μg/ml	53.60 ± 1.95*	37.83 ± 3.18	8.57 ± 1.77*	46.40 ± 1.95*
300 μg/ml	64.59 ± 3.38*	24.64 ± 5.35*	10.77 ± 8.17	35.41 ± 3.38*

Data shown as means ± standard deviation (n = 3). OD: optical density. *P < 0.05 versus the control group.

### Proteomic analysis revealed that cell cycle inhibition was involved in protosappanin B-induced cell apoptosis

**Figure 5:**
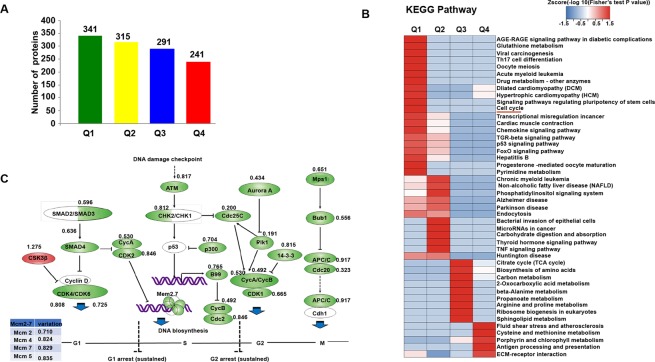
Impact of protosappanin B on the proteomics of bladder cancer cells. (**A**) The proteomic analysis identified 4914 proteins that were changed by protosappanin B treatment. Among them, 3949 proteins were quantified. The differentially expressed proteins were grouped as: Q1 (0 < ratio ≤ 1/1.5), Q2 (1/1.5 < ratio ≤ 1/1.3), Q3 (1.3 < ratio ≤ 1.5) and Q4 (ratio > 1.5). (**B**) The proteins in each group were analyzed using the Kyoto Encyclopedia of Genes and Genomes (KEGG) and subjected to cluster evaluation. (**C**) Cell cycle-related proteins were significantly affected by protosappanin B.

The proteomic analysis was used to identify potential altered proteins. Among the 4914 identified proteins, the proteins both appeared in the vehicle and protosappanin B treated groups were quantified and compared (3494), and their intensity was measured as follows: intensity ratio = Protosappanin B treated group/vehicle group. The differentially expressed proteins were grouped as: Q1 (0 < ratio ≤ 1/1.5), Q2 (1/1.5 < ratio ≤ 1/1.3), Q3 (1.3 < ratio ≤ 1.5) and Q4 (ratio > 1.5) (Fig. 5A). Then, the proteins in each group were analyzed using the Kyoto Encyclopedia of Genes and Genomes (KEGG) and subjected to cluster evaluation. As shown in Fig. 5B,C, cell cycle-related proteins were significantly affected by protosappanin B. These findings further support that cell cycle arrest was, at least in part, involved in protosappanin B-induced cell apoptosis.

## Discussion

The main finding of the present study was that protosappanin B inhibited the proliferation and promoted the apoptosis of two human bladder cancer cell lines (T24 and 5637 cells) in a concentration-dependent manner. Furthermore, in both cell types, protosappanin B caused arrest of the cell cycle in the G_1_ phase. These data suggest that protosappanin B may have an anti-tumor effect against bladder cancer and that the mechanism may involve interference with cell cycle regulation, preventing transition of cells from G_1_ to S-phase, as suggested by the proteomics analysis.

Su Fu’ning Lotion, which is approved for medical use in China, has been shown to reduce the recurrence rate of bladder cancer^34^. The active ingredient of Su Fu’ning Lotion is a Lignum Sappan extract that contains protosappanin B and brazilin^35^, which are polyphenols with similar structures and chemical properties. In previous research by our group, we found that protosappanin B can inhibit the proliferation of four types of tumor cell, including BTT, T24, Hela, and SW480 cells^36,37^. Furthermore, protosappanin B has been shown to have anti-tumor effects *in vivo* and *in vitro*^33^. Nevertheless, there are very few published studies on the anti-tumor actions of protosappanin B. Therefore, the present *in vitro* study was designed to test the anti-tumor effects of a range of protosappanin B concentrations on two bladder cancer cell lines, T24 cells (representing invasive bladder cancer) and 5637 cells (representing high-risk superficial bladder cancer). In addition, cell cycle analysis was undertaken to explore the mechanism of action.

In the initial experiments, we observed the morphological changes in T24 and 5637 cells exposed to 200 μg/mL protosappanin B for 24 h. The results indicated that both types of bladder cancer cells showed morphological abnormalities following treatment with protosappanin B, with evidence of cell lysis. Furthermore, the MTT assay revealed that protosappanin B (applied for 48 h) significantly inhibited the growth and proliferation of T24 and 5637 cells in a concentration-dependent manner, with particularly powerful effects at 200 µg/mL. These findings are consistent with a previous study reporting that protosappanin B reduced the viability of bladder cancer BTT cells as well as human colon cancer cells (HCT-116 and SW-480)^33^. In the present study, the IC50 values for cell growth inhibition by protosappanin B were 82.78 µg/mL in T24 cells and 113.79 µg/mL in 5637 cells. These values are broadly similar to the value of 76.53 µg/mL in BTT bladder cancer cells reported previously^33^. The IC50 values in SW-480 and HCT-116 cells were 21.32 µg/mL and 26.73 µg/mL^33^, respectively, suggesting that different types of cancer cell may have different sensitivity to protosappanin B. Our observations are similar to those of other studies showing that Lignum Sappan extract or brazilin inhibits the proliferation of various cancer cell lines^16–19,22,24^.

Apoptosis is the main mechanism by which anti-cancer drugs lead to cell death. In the present study, protosappanin B (100–300 µg/mL) was found to promote the apoptosis of T24 and 5637 cells in a concentration-dependent manner, which would be consistent with the results of the MTT assay. Therefore, we speculate that the inhibitory effect of protosappanin B on cell viability was associated with the induction of apoptosis. A previous investigation also showed that protosappanin B could induce the apoptosis of bladder cancer cells (T24 and BTT), although a much higher concentration (2 mg/mL) was used^33^. In addition, similar pro-apoptotic effects of Lignum Sappan extract and brazilin have been reported^18–21,23–25^. We investigated the effect of protosappanin B on Bax and Bcl-2 protein expression. Bcl-2 is an anti-apoptotic protein. On the other hand, Bax forms a heterodimer with Bcl-2 to activate apoptosis^38^. In the present study, protosappanin B decreased Bcl-2 protein levels and increased Bax protein expression, leading to the increased apoptosis of bladder cancer cells. Of course, this is only a glimpse of the mechanisms responsible for decreased cell growth and increased apoptosis caused by protosappanin B. Additional study is necessary to determine the mechanisms of action comprehensively.

Uncontrolled proliferation of cells caused by dysregulation of the cell cycle is one of the characteristics of tumor cells. Therefore, induction of cell cycle arrest is an important mechanism of action for many anti-cancer drugs. When stimulated by proliferative signals, cells enter into the cell cycle (G_1_ to S to G_2_ to M phases) from a stationary state (G_0_ phase). The cell cycle is regulated at two transition points, G_1_/S (between the end of the G_1_ phase and the S phase) and G_2_/M (in the M phase). The G_1_/S checkpoint is a limiting step in the cell cycle; if the cycle is arrested at this point, the proliferation of cells can be effectively limited^39^. In the present study, protosappanin B caused a concentration-dependent reduction in the proportion of T24 and 5637 cells in the S-phase and an increase in the proportion of cells in the G_1_-phase. Therefore, protosappanin B arrested cells in the G_1_ phase, decreasing the proliferative index. It is likely that protosappanin B arrests progression from the G_1_ phase to the S phase by intervening at a regulatory point in the cell cycle. Interestingly, a previous study in head and neck cancer cell lines found that Lignum Sappan extract increased the number of cells in the sub-G1 phase of the cell cycle, an effect that was associated with increased cellular levels of p53 and p21^17^. Another study reported that Lignum Sappan extract up-regulated the mRNA transcription of P16 and Rb1 in lung cancer cells and caused cell cycle arrest at the G0/G1 and S phases^40^. It is possible that the various components of Lignum Sappan have different effects on cell cycle regulation, since sappanchalcone was found to induce G2/M arrest^18^. Additional research is needed to clarify the detailed mechanisms by which protosappanin B arrests bladder cancer cells in the G_1_ phase and causes cell apoptosis.

## Conclusions

Protosappanin B inhibits the proliferation of T24 and 5637 cells and induces their apoptosis. Furthermore, these effects are related to arrest of the cell cycle in the G_1_ phase. Additional studies are needed to identify the mechanisms by which protosappanin B elicits these actions. Nonetheless, the present study suggests that protosappanin B may potentially be an effective and natural drug for the treatment of bladder cancer.

## Methods

### Cell culture

The human bladder cancer cell line T24 was purchased from the Shanghai Institutes for Biological Sciences (Chinese Academy of Sciences, Shanghai, China). The human bladder cancer cell line 5637 was purchased from the Cell Bank of the Chinese Academy of Sciences. The normal uroepithelium cell line SV-HUC-1 was purchased from the American Type Culture Collection (ATCC; Manassas, VA, USA). The T24, 5637, and SV-HUC-1 cells were cultured in RPMI 1640 medium (GIBCO, Thermo Fisher Scientific, Waltham, MA, USA) containing 0.05 g/L streptomycin, 0.05 g/L penicillin, 0.8 g/L NaHCO_3_, 3.6 g/L HEPES, and 10% fetal bovine serum (Hangzhou Sijiqing Biotechnology Materials Co., Ltd, Hangzhou, China). Cell culture was performed in a thermostatic incubator (NU-5500E; NuAire, Plymouth, MN, USA) at 37 °C with 5% CO_2_ and saturated humidity. Cell suspensions were prepared by digesting the cells in the logarithmic growth phase with 0.25% trypsin (Shanghai Biotech Bioengineering Co., Ltd, Shanghai, China).

### Cell morphology

T24 cells (4 × 10^5^ cells/well) or 5637 cells (5 × 10^5^ cells/well) were transferred to a 6-well plate. Protosappanin B was added to three of the wells (final concentration, 200 μg/mL), and an equal volume of culture medium was added to the other three wells (as a control group). Protosappanin B (purity >96.7%) was isolated and purified from Lignum Sappan (a TCM) by Professor Zhang Shengwan at the College of Life Sciences, Shanxi University, China^33^. Protosappanin B is used in clinics at 0.5 mg/kg and the concentration for *in vitro* experiments was tested by preliminary experiments. Changes in cell morphology were observed at 24 h (DMIL090-135.001 inverted microscope; Leica, Wetzlar, Germany) and compared among the control and protosappanin B groups.

### Cell proliferation

Cell proliferation was assessed using the methyl thiazolyl tetrazolium (MTT) assay. T24 cells were seeded in a 96-well plate at 4 × 10^3^ cells/well, 5637 cells were seeded in a 96-well plate at 9 × 10^3^ cells/well, and SV-HUC-1 cells were seeded in a 96-well plate at 4 × 10^3^ cells/well. After the addition of culture medium (100 µL/well), the plates were cultured in an incubator (NU-5500E; NuAire) at 37 °C with 5% CO_2_ for 24 h. The original culture medium was then discarded, and 200 µL of culture medium containing different concentrations of protosappanin B (final concentrations: 12.5, 25, 50, 100, and 200 μg/mL) were added to the cells in the protosappanin B groups (5 wells/group). The same volume of medium without protosappanin B was added to the cells in the control group (5 wells). Culture medium without cells was used as the zero group (5 wells). After 48 h, 20 µL of 5 mg/mL MTT solution (Solarbio, Beijing, China) were added to each well and the cells were cultured for a further 4 h. The culture medium was then carefully removed, 150 µL of dimethyl sulfoxide (Tianjin Fuyu Fine Chemical Co., Ltd., Tianjin, China) were added to each well, and the plate was placed on a shaking incubator (low speed) for 10 min to fully dissolve the crystals. The absorbance value, i.e. optical density (OD) value, was measured at 570 nm using a microplate reader (SunRise microplate reader; Tecan Corporation, Grödig, Austria). The growth inhibition rate (%) for each cell line was calculated as [1 − (average OD value in experimental group/average OD value in control group)] × 100%. The concentration of protosappanin B producing 50% inhibition of cell growth (IC_50_ value) was calculated as log[IC_50_] = log[maximum dose] − log[maximum dose/relative dose](sum of positive reaction rates − (3 − maximum positive reaction rate − minimum positive reaction rate)/4).

### Cell apoptosis

Cell apoptosis was detected using an annexin-V/propidium iodide (PI) assay (Bioengineering (Shanghai) Co., Ltd., Shanghai, China). T24 cells (4 × 10^5^ cells/well) and 5637 cells (5 × 10^5^ cells/well) were cultured in 6-well plates for 24 h. The supernatant was discarded and culture medium containing different concentrations of protosappanin B was added (final concentration: 100, 150, 200, or 300 μg/mL). An equal volume of culture medium not containing protosappanin B was used as a negative control. After 24 h of culture, the cells were digested, collected, and centrifuged (KDC-2044 low-speed refrigerated centrifuge; USTC ChuangXin Co., Ltd., Anhui, China) at 1000 r/min for 5 min. The supernatant was discarded and the cells were re-suspended in phosphate-buffered saline (PBS). The cells were filtered through a nylon sieve, centrifuged (1000 r/min, 5 min), and the supernatant was discarded. The cells were re-suspended in binding buffer and 5 μL of the apoptosis detection reagents (annexin-V-fluorescein isothiocyanate and PI) were added. The cells were incubated at room temperature in the dark for 15 min. The apoptosis of T24 and 5637 cells was detected using flow cytometry (FACSCalibur flow cytometer, BD Biosciences, San Jose, CA, USA), with the experiments performed in triplicate. In the scatterplots, normal live cells (annexin-V-negative and PI-negative) were shown in the lower left quadrant, early apoptotic cells (annexin-V-positive and PI-negative) were shown in the lower right quadrant, and late apoptotic cells (annexin-V-positive and PI-positive) were shown in the upper right quadrant. The total apoptosis rate was calculated as the sum of the early and late apoptosis rates.

### Western blot

T24 and 5637 cells were treated with 0, 100, 200, or 300 µg/ml protosappanin B for 48 h. Cells were lysed using the RIPA buffer (Beyotime Institute of Biotechnology, Haimen, China). Protein concentration was assayed using the Bradford assay. Proteins (20 µg/lane) were separated by polyacrylamide gel electrophoresis (Bio-Rad, Hercules, CA, USA) and transferred to PVDF membranes (Millipore corp., Billerica, MA, USA). The primary antibodies against Bax (1:200; Abcam, Cambridge, MA, USA) and Bcl-2 (1:200; Abcam, Cambridge, MA, USA) were incubated overnight at 4 °C. The membranes were washed and incubated for 2 h with the HRP-labelled secondary antibody (Abcam, Cambridge, MA, USA). Protein bands were revealed using the ECL reagent (Pierce Chemical, Dallas, TX, USA). The blots were scanned and analyzed using the Image J software (National Institutes of Health, Bethesda, MD, USA).

### Cell cycle analysis

T24 cells (8 × 10^5^ cells/well) and 5637 cells (1 × 10^6^ cells/well) were cultured in 6-well plates for 24 h. The supernatant was discarded and culture medium containing different concentrations of protosappanin B was added (final concentration: 100, 150, 200, 250, or 300 μg/mL). An equal volume of culture medium without protosappanin B was used as a negative control. After 48 h of culture, the cells were digested, collected, and centrifuged (1000 r/min, 5 min). The supernatant was discarded and the cells were washed with PBS and fixed in 70% cold ethanol overnight at 4 °C. The cells were washed with PBS, filtered through a nylon sieve, and centrifuged (1000 r/min, 5 min), and the supernatant was discarded. The DNA content was examined using a cell cycle detection kit (KGA512; KeyGen Biotechnology, Nanjing, China) and analyzed using flow cytometry (FACSCalibur flow cytometer). Each sample was analyzed in triplicate.

### Proteomics analysis

Proteomic analyses were performed to explore the possible mechanisms involved in the inhibition of cell growth by protosappanin B.

Protein extraction: After T24 cells were treated with protosappanin B or corresponding vehicle for 48 h, the cells were collected and the proteins were extracted. The cells were sonicated three times on ice in lysis buffer (8 M urea, 1% protease inhibitor cocktail). The debris was removed by centrifugation at 12,000 × g at 4 °C for 10 min. The supernatant was collected and the protein concentration was determined by the BCA method (Beyotime, Beijing, China).

Trypsin digestion: For digestion, the protein was reduced with 5 mM dithiothreitol for 30 min at 56 °C and alkylated with 11 mM iodoacetamide for 15 min at room temperature in the dark. The protein sample was diluted with 100 mM NH_4_HCO_3_ to urea concentration (2 M). Finally, trypsin was added at a 1:50 trypsin-to-protein mass ratio for the first digestion overnight and 1:100 trypsin-to-protein mass ratio for a second 4 h-digestion.

High-performance liquid chromatography (HPLC) fractionation: The tryptic peptides were fractionated into fractions by high pH reverse-phase HPLC using an Agilent 300 Extend C18 column (5 μm particles, 4.6 mm ID, 250 mm length). Briefly, peptides were first separated with a gradient of 8% to 32% acetonitrile (pH 9.0) over 60 min into 60 fractions. Then, the peptides were combined into 4 fractions and dried by vacuum centrifuging.

LC-MS/MS analysis: The tryptic peptides were dissolved in 0.1% formic acid (solvent A), directly loaded onto a home-made reversed-phase analytical column (15-cm length, 75 μm ID). The gradient was comprised of an increase from 6% to 23% solvent B (0.1% formic acid in 98% acetonitrile) over 26 min, 23% to 35% in 8 min and climbing to 80% in 3 min then holding at 80% for the last 3 min, all at a constant flow rate of 400 nl/min on an EASY-nLC 1000 UPLC system. The peptides were subjected to NSI source followed by tandem mass spectrometry (MS/MS) in Q ExactiveTM Plus (Thermo Fisher Scientific, Waltham, MA, USA) coupled online to the UPLC. The electrospray voltage was 2.0 kV. The m/z scan range was 350 to 1800 for full scan, and intact peptides were detected in the Orbitrap at a resolution of 70,000. Peptides were then selected for MS/MS using NCE setting of 28 and the fragments were detected in the Orbitrap at a resolution of 17,500. A data-dependent procedure alternated between one MS scan followed by 20 MS/MS scans with 15.0-s dynamic exclusion. Automatic gain control (AGC) was set at 5E4.

Enrichment-based clustering: For hierarchical clustering based on enrichment of pathway analysis, we first collated all the categories obtained after enrichment along with their P values, and then filtered for those categories which were at least enriched in one of the clusters with P < 0.05. This filtered P value matrix was transformed by the function x = −log10 (P value). Finally, those x values were z-transformed for each functional category. Those z scores were then clustered by one-way hierarchical clustering (Euclidean distance, average linkage clustering) in Genesis. Cluster membership were visualized by a heat map using the “heatmap.2” function from the “gplots” R package.

### Statistical analysis

Statistical analysis was performed using SPSS 17.0 (IBM, Armonk, NY, USA). The data were expressed as means ± standard deviation (SD). Comparisons among multiple groups were made using one-way analysis of variance (ANOVA) with the LSD-t (homogeneity of variance) or Dunnett’s T3 (heterogeneity of variance) post-hoc test. *P* < 0.05 was considered to indicate statistical significance.

## Supplementary information

supplement info

## Data Availability

The datasets used and/or analysed during the current study are available from the corresponding author on reasonable request.
