# Supplementary material for: Protosappanin B promotes apoptosis and causes G1 cell cycle arrest in human bladder cancer cells
Source: Sci Rep. 2019 Jan 31;9:1048. doi: 10.1038/s41598-018-37553-z (PMC6355918; doi:10.1038/s41598-018-37553-z)

Protosappanin B promotes apoptosis and causes G_1 cell cycle arrest in human bladder cancer cells

Running title: Anti-tumor effect of protosappanin B in bladder cancer cells

Xihua Yang[1,†], Lili Zhao[1,†], Tingting Zhang[2], Junfeng Xi[1], Shuze Liu[3], Liansheng Ren[1,*], Yaqin Zheng[1,*], Huanhu Zhang[1,*]

[1]Affiliated Cancer Hospital, Shanxi Medical University, Taiyuan 030001, China

[2]Research Institute of Applied Biology, Shanxi University, Taiyuan 030006, China

[3]Department of Computer Science, Rensselaer Polytechnic Institute, Troy, 12180, USA

[†]These authors contributed equally to this work.

[*]Corresponding Author:

Huanhu Zhang

Affiliated Cancer Hospital, Shanxi Medical University, Taiyuan 030001, China

Email: zhhh31@163.com

Tel: +86-13403419805

Liansheng Ren

Affiliated Cancer Hospital, Shanxi Medical University, Taiyuan 030001, China

Email: liansheng.ren@163.com

Yaqin Zheng

Affiliated Cancer Hospital, Shanxi Medical University, Taiyuan 030001, China

E-mail:sxzhengyaqin@163.com

bcl-2-1

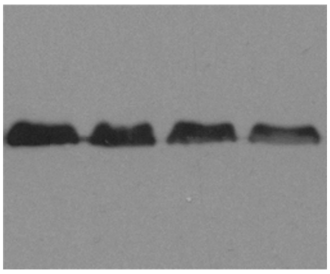

bcl-2-2

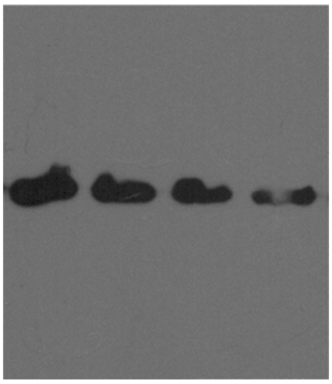

bax-1

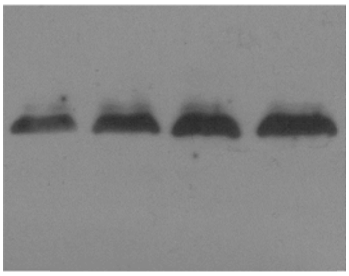

bax-2

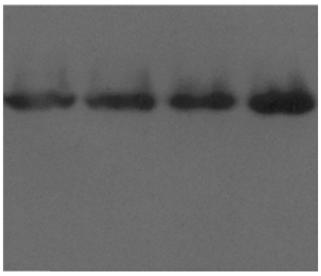

beta-actin-1

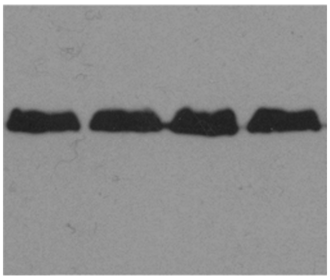

beta-actin-2

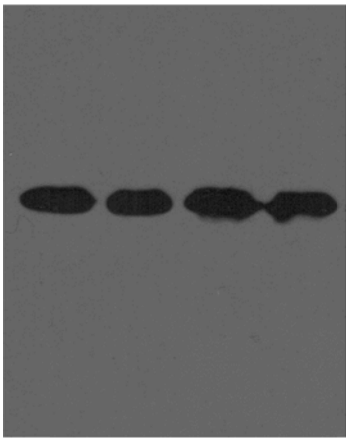

Supplement: Supplementary file 1 — supplement info [file 41598_2018_37553_MOESM1_ESM.pdf]
